# Case series multilocular cystic hemangioma of the liver: Three cases and literature review

**DOI:** 10.1097/MD.0000000000039287

**Published:** 2024-08-16

**Authors:** Chunmiao Liang, RiSheng Yu, Liuhong Wang, Ying Chen

**Affiliations:** a Department of Radiology, Shengzhou People’s Hospital (Shengzhou Branch of the First Affiliated Hospital of Zhejiang University School of Medicine), Shengzhou, Zhejiang, P.R.China; b Department of Radiology, Second Affiliated Hospital of Zhejiang University School of Medicine, Hangzhou, China.

**Keywords:** CT, hemangioma, MR, multilocular cystic

## Abstract

**Rationale:**

Multilocular cystic hemangioma is a rare benign tumor classified as an atypical hemangioma. Currently, there are limited imaging reports available, and the imaging characteristics can be challenging to distinguish from other malignant multilocular cystic liver diseases such as cystadenocarcinoma, necessitating confirmation through pathological diagnosis. Here, we discuss the imaging features of 3 cases of multilocular cystic hemangiomas.

**Patient concerns and Diagnoses:**

Case 1 was a 24-year-old young female, and Case 2 involved a 60-year-old elderly male. Both patients were asymptomatic and physical examination revealed hepatic space-occupying lesions. Imaging findings revealed multilocular cystic lesions in the left liver with septa, calcification, a high diffusion-weighted magnetic resonance imaging (DWI) signal at the edge of the lesion, and progressive enhancement of the cyst wall and septa. Case 3 involved a 50-year-old male patient with epigastric distending pain for 1 month and sudden severe abdominal pain for 14 hours. Imaging results revealed a multilocular cystic lesion in the left liver with septa and tumor bleeding, a high DWI signal, and an enhanced cyst wall and septa. The pathological diagnosis confirmed a hepatic hemangioma.

**Interventions:**

All 3 patients underwent liver tumor resection.

**Outcomes:**

All 3 patients recovered successfully without any intraoperative or postoperative complications during the follow-up periods of 5 years, 6 months, and 5 months. There were no signs of recurrence.

**Lessons:**

Liver imaging revealed multilocular cystic lesions with features, such as compartmentalization, calcification, or bleeding. Multilocular cystic hemangiomas should be considered in imaging diagnosis. Enhancing our understanding of multilocular cystic hemangiomas can aid in improving the differential diagnosis of other malignant multilocular cystic liver diseases, ultimately reducing unnecessary liver resection.

## 1. Introduction

Hepatic hemangioma is the most common benign liver tumor, with cavernous hemangioma being the predominant clinical variant. Most hepatic hemangiomas exhibit typical features on imaging. Nevertheless, a minority of hepatic hemangiomas display atypical imaging features, which can lead to misdiagnosis, particularly when confused with certain liver malignancies. In such cases, surgical pathology is necessary to confirm diagnosis, potentially resulting in unnecessary surgical procedures. Among atypical hemangiomas, multilocular cystic hemangiomas are rare with limited reports of relevant imaging studies, primarily isolated case reports. In this report, we present 3 cases of multilocular cystic hemangiomas with pathologically confirmed diagnoses, showing compartmentalized imaging findings across ultrasound (US), computed tomography (CT), and magnetic resonance (MR) imaging. Additionally, we offer a comprehensive review and summary of all reported cases found in the relevant Chinese and English language literature.

## 2. Case presentation

Case 1 was a 24-year-old female patient who was admitted to the hospital a month prior to an ultrasound examination that revealed a liver space-occupying lesion. After admission, the patient was asymptomatic and exhibited no signs of distress. Laboratory tests, including liver function and tumor markers such as carbohydrate antigen 199 (CA199), carbohydrate antigen 125 (CA125), carcinoembryonic antigen (CEA), and alpha-fetoprotein (AFP), all yielded results within the normal range.

**Figure 1.**
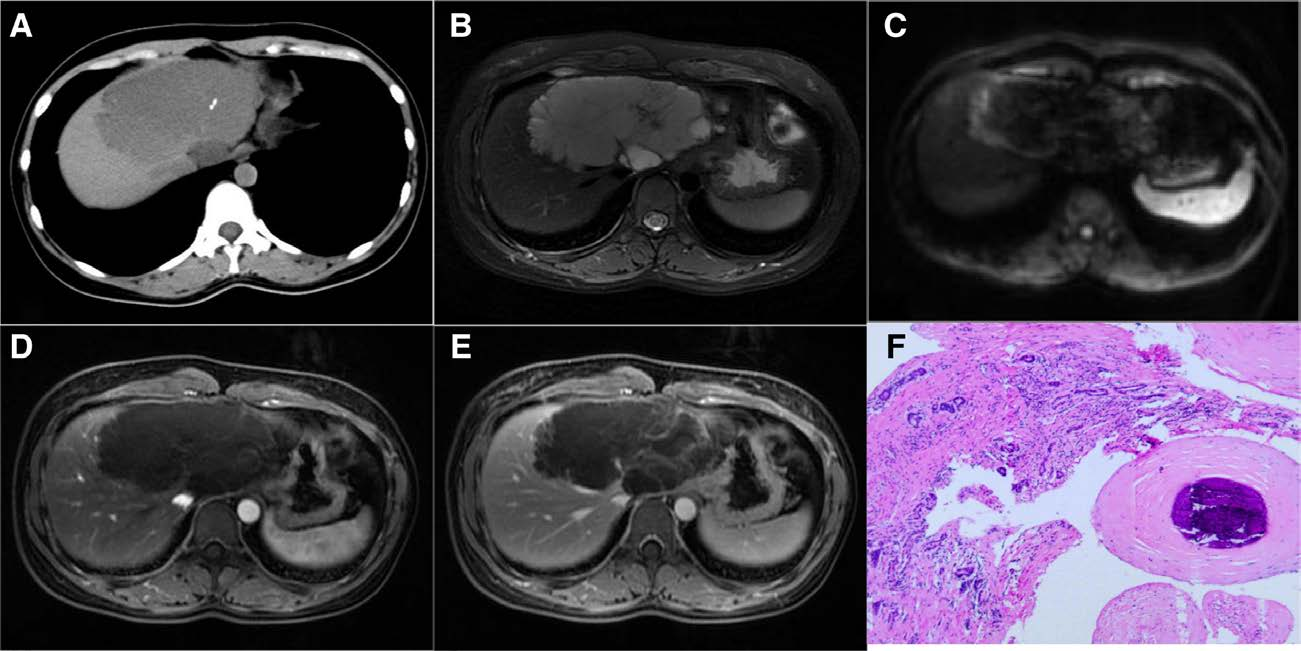
(A) A plain CT scan revealed a low-density mass in the left liver, measuring approximately 11.7 × 6.8 cm in size, with localized short strips of calcification. (B) T2WI lesions displayed slightly higher signals, with increased signals around the perimeter, low signal separations, and well-defined boundaries. (C) DWI exhibited a high signal at the lesion’s edge. (D and E) Enhanced MR imaging in the arterial phase and delayed phase demonstrated progressive enhancement of the cyst wall and septa, with no enhanced wall nodules. (F) HE staining indicated the presence of malformed blood vessels. CT = computed tomography, DWI = duffusion weighted imaging, HE = hematoxylin and eosin, T2WI = T2-weighted imaging.

Abdominal enhanced CT revealed a multilocular cystic lesion in the left liver with septa and calcification (Fig. 1A), along with progressive enhancement of the cyst wall and septa. Liver-enhanced MR imaging revealed a multilocular cystic lesion in the left hepatic region with star-shaped divisions, T2WI lesions displayed slightly higher signals (Fig. 1B), a high duffusion weighted imaging (DWI) signal at the edge (Fig. 1C), and progressive enhancement of the cyst wall and divisions (Fig. 1D and E). In combination, these imaging findings raised the suspicion of hepatic cystadenoma with malignant transformation.

Following laparotomy of the liver tumor under general anesthesia, pathological diagnosis (Fig. 1F) indicated the presence of abnormal blood vessels in the liver tissue, accompanied by focal calcification. This led to the consideration of vascular developmental malformations and hemangiomas as underlying conditions.

Case 2 was a 60-year-old male patient who was hospitalized 2 months prior to a routine physical examination that revealed a space-occupying lesion in the liver. The patient remained asymptomatic after admission, and showed no signs of discomfort or illness. Laboratory tests consistently showed normal liver function and tumor marker levels within the reference range.

**Figure 2.**
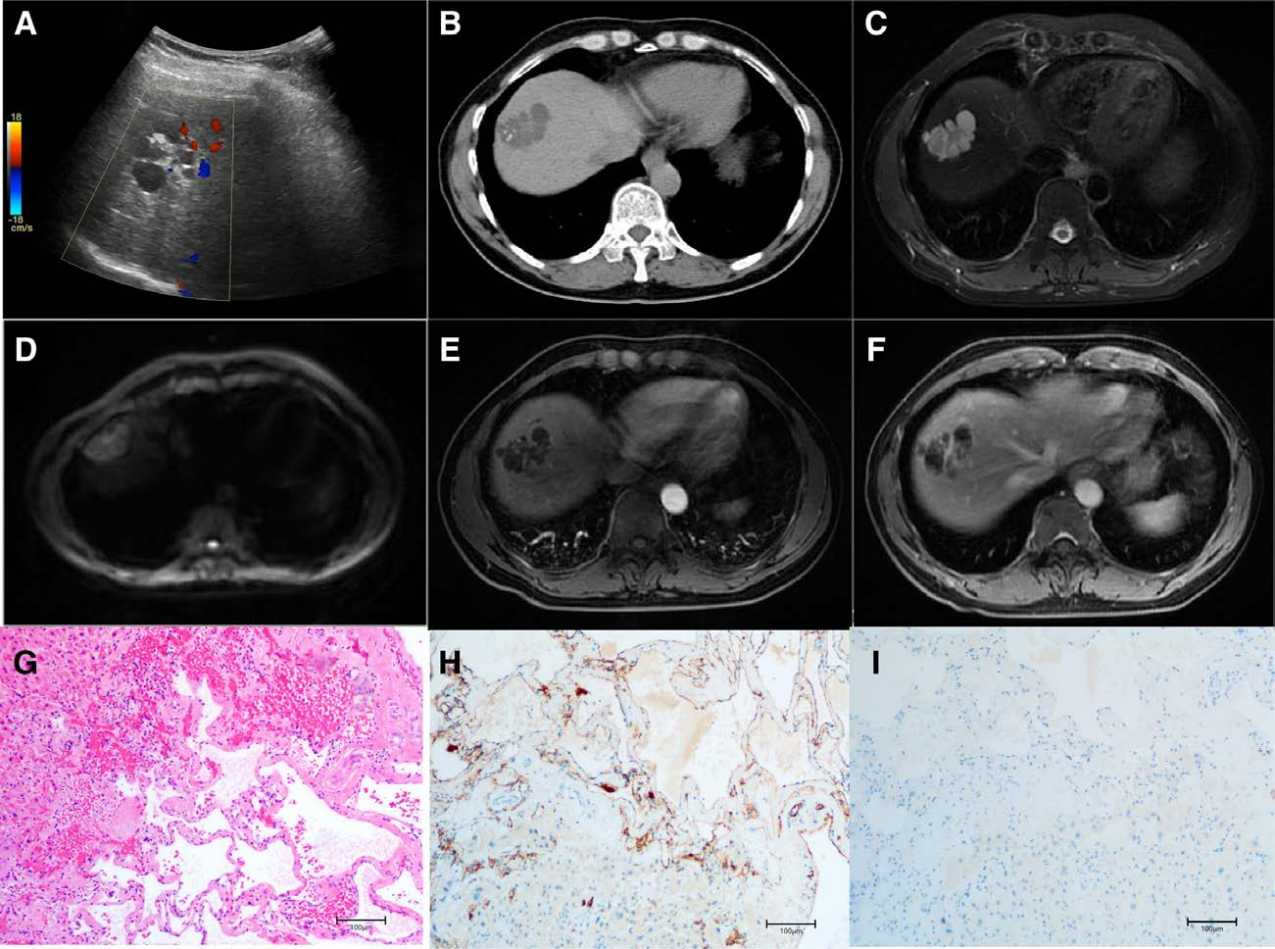
(A) Ultrasound examination revealed a liver polycystic space-occupying lesion with a blood flow echo. (B) A plain CT scan displayed multilocular cystic lesions with compartments in segment VIII of the liver, approximately 4.2*3.4 cm in size, featuring speckled calcification. (C) T2WI demonstrated high signal intensity, clear boundaries, and lobulation. (D) Diffusion-weighted imaging (DWI) exhibited a high signal at the lesion’s edge. (E and F) Enhanced MR images from the arterial phase and delayed phase illustrated progressive enhancement of the cyst wall and septa, with no enhanced wall nodules. (G) Hematoxylin and eosin staining (HE ×10) revealed multilocular cystic masses in the liver. (H) CD31 staining (×10 magnification) showed a positive result. (I) D2-4 staining (×10 magnification) produced a negative result. CT = computed tomography, DWI = duffusion weighted imaging, HE = hematoxylin and eosin, T2WI = T2-weighted imaging.

US examination (Fig. 2A) revealed a multicystic mass located in segment VIII of the liver, initially raising concerns regarding the possibility of liver cystadenoma. Subsequent abdominal enhanced CT scans (Fig. 2B) confirmed the presence of multilocular cystic lesions within segment VIII of the liver that exhibited characteristic septa and calcifications. Notably, progressive enhancement of both the cyst wall and the septa was observed.

Further evaluation using liver-enhanced MR (Fig. 2C) depicted a star-shaped division pattern within the multilocular cystic lesion in segment VIII. Additionally, a high DWI signal intensity was detected at the edge (Fig. 2D), along with a progressive enhancement pattern involving the cyst wall and divisions (Fig. 2E and F). These collective imaging findings raised the suspicion of focal bile duct hamartoma or low-grade malignant liver tumor.

Laparoscopic partial hepatectomy was performed to definitively diagnose the patient’s condition and to provide appropriate treatment. Subsequent pathological examination of the resected tissue confirmed the presence of a multilocular cystic mass in the liver accompanied by focal calcification (Fig. 2G–I). Immunohistochemical analysis, which included CD31 and D2-4 markers, conclusively identified the lesion as hemangioma.

In Case 3, a 50-year-old middle-aged male patient presented with a history of epigastric distension lasting for 1 month, along with sudden severe abdominal pain experienced over a 14-hour period. Laboratory examination results revealed a D-dimer level of 4070 µg/L (FEU), while tumor markers remained within the normal range.

**Figure 3.**
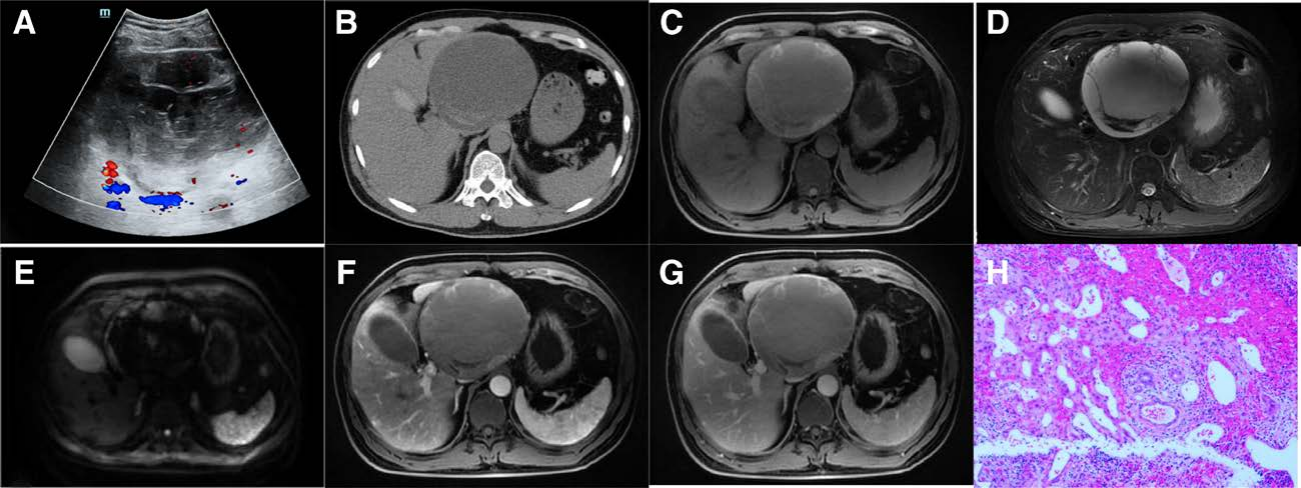
(A) Ultrasonography revealed a left hepatic cystic lesion characterized by multiple compartments and a flocculent echo. (B) A plain CT scan identified a multilocular cystic lesion with compartmentalization in the left liver, measuring approximately 13.1*12.1 cm. Notably, a high-density lesion was observed at the edge of the lesion, indicative of bleeding. (C and D) T1WI and T2WI demonstrated mixed signals at the lesion’s edge, suggesting different stages of bleeding. The lesions appeared compartmentalized, with well-defined boundaries. (E) DWI displayed a high signal intensity at the lesion’s edge. (F and G) Enhanced MR imaging during both the arterial phase and delayed phase exhibited progressive enhancement of the cyst wall and septa. (H) HE staining confirmed the presence of bleeding within the lesion. CT = computed tomography, DWI = duffusion weighted imaging, HE = hematoxylin and eosin, T1WI = T1-weighted imaging, T2WI = T2-weighted imaging.

Initial ultrasonography (Fig. 3A) revealed a solid left hepatic mass characterized by multiple compartments and a flocculent echo. This appearance was suggestive of tumor rupture and bleeding. A subsequent abdominal CT (Fig. 3B) confirmed the presence of a left-sided cystic space-occupying lesion with signs of bleeding.

Upon further evaluation using liver MR (Fig. 3C–G), a multilocular cystic lesion with compartmentalization was identified in the left liver. The lesion exhibited mixed signals and measured approximately 13.1*12.1 cm in size. Notably, a high signal intensity on DWI was observed at the edge of the lesion, along with enhancement of both the cyst wall and compartments during the post-contrast phases.

On the basis of the imaging findings, a mucous cystic tumor with concurrent bleeding was suspected. To obtain a definitive diagnosis and provide necessary treatment, the patient underwent laparoscopic partial hepatectomy. Subsequent pathological examination (Fig. 3H) of the resected tissue revealed hepatic fibrocystic wall tissue with a focal lining composed of a single or multilayer cubic epithelium. The specimen showed significant chronic inflammatory cell infiltration, bleeding, and localized hemangiomatous hyperplasia.

## 3. Discussion

The pathological mechanisms underlying hepatic hemangiomas remain unclear. It is currently believed that hepatic hemangioma is a type of vascular malformation that typically lacks an envelope. It primarily consists of small blood vessels dilated at the end of the hepatic artery and numerous sac-like dilated blood sinuses. The growth of these structures is driven by further expansion under the influence of blood flow. Fibrous septa within hemangiomas are primarily composed of fibroblasts and collagen fibers, whereas the blood sinuses and small vascular lumens feature complete endothelial cells.^[1]^ Based on the morphological characteristics observed on pathology, most liver hemangiomas display distinct enhancement patterns following intravenous administration of the contrast material. These patterns are characterized by hypoattenuating lesions on non-enhanced images, followed by peripheral globular enhancement of the lesion on arterial-phase images, progressive hyperattenuation from the periphery to the center, and persistent enhancement on delayed-phase images. This centripetal enhancement pattern is referred to as the “fill-in phenomenon.”^[2]^ Therefore, a typical imaging examination of hemangiomas is usually sufficient to confirm the diagnosis, eliminating the need for further punctures or surgeries to confirm pathological results.

However, many hemangiomas exhibit atypical features that can lead to misdiagnosis and confusion with other liver diseases. Vilgrain et al published an article describing the imaging findings of less common atypical hemangiomas, such as large heterogeneous giant hemangiomas, rapidly filling hemangiomas, calcified hemangiomas, hyalinized hemangiomas, multilocular cystic hemangiomas, hemangiomas with fluid-fluid levels, and pedunculated hemangiomas.^[3]^ According to Valerie Vilgrain et al, our case can be classified as multilocular cystic hemangioma. We conducted a literature search of all cases of multilocular cystic hemangioma published in Chinese and English literature, and a total of 9 cases were collected, including 3 in this study, to summarize the clinical manifestations, pathology, and imaging features of multilocular cystic hemangioma (Table 1).

**Table 1:** Summary of multilocular cystic hemangioma.

Case	Literature	Age/sex	Symptom	Size (cm)	Location	Gross findings	Imaging features
1	Wang S et al (2023)^[4]^	54/Female	abdominal pain	3.4*3.2	S7	Multilocular cystic structure; Microscopically, the tumor tissue was composed of enlarged lumen of different sizes, some lumen could be seen in powder fluid, and some lumen could be seen in red blood cells	Multilocular cystic structure with septa, clear boundary, lobulated lesions, calcification, cyst wall and septa enhancement
2	Qiu LH et al (2006)^[5]^	23/Female	abdominal distension	18*13	Left liver	The structure of the multilocular cystic cavity is full of bloody fluid, and the inner wall of the cystic cavity is reticulated	Multilocular cystic structure with septa, clear lesion boundary, lobulated, cyst wall and septa enhancement
3	Scribano E et al (1996)^[6]^	44/Female	Diffuse abdominal pain	Greater than 4	Left liver	Multilocular cystic structure with local calcification	Multilocular cystic structure with septa, clear lesion boundary, lobulated, cyst wall and septa reinforced, abdominal hematoma
4	Cha EY et al (2008)^[7]^	62/Female	Asymptomatic	6.3*5.7	S4/7/8	Multilocular cystic structure and septa, full of necrotic matter, serous fluid, or thrombus, with a red central region; The multilocular cyst wall is lined with endothelial cells	Multilocular cystic with compartmentalization, clear boundary, lobulated margin, unstrengthened cystic part, progressive enhancement of cyst wall and compartmentalization; High signal in T1 sequence at the edge of the lesion
5	Hihara T et al (1990)^[8]^	61/Female	Abdominal pain	Greater than 4	S2/3/4	Multilocular cystic structure with calcification, containing yellow serous fluid	Multilocular cystic with compartmentalization, clear boundary, lobulated margin, reinforced cyst wall, and compartmentalization
6	Lee NK et al (2022)^[9]^	48/Female	Asymptomatic	14*14	S5/6	Multilocular cystic mass, including fluid, necrosis, and bleeding; The capsule wall is lined with flat or cube endothelial cells	Multilocular cystic with compartmentalization, clear boundary, lobed margin, cyst wall, and compartmentalization gradually strengthened; Some lumen have high T1 signal and low T2 signal focus
7	Text case1	24/Female	Asymptomatic	11.7*6.8	S4/8	Multilocular cystic structure with local calcification	Multilocular cystic with compartmentalization, clear boundary, marginal lobed, calcification, progressive strengthening of the cyst wall, and compartmentalization
8	Text case2	60/Male	Asymptomatic	4.2*3.4	S8	Multilocular cystic structure with local calcification	Multilocular cystic companion asteroid septum, calcification, progressive strengthening of the cyst wall and septum
9	Text case3	50/Male	Severe abdominal pain	13.1*12.1	Left liver	Fibrous capsular wall tissue, capsular wall is focally lined with a single or multiple layer of cubic epithelium, accompanied by a large number of chronic inflammatory cell infiltration and bleeding, local vascular hyperplasia	Multilocular cystic with septa, enhanced cyst wall and septa, and bleeding

Based on a review of published cases, the age of onset of multilocular cystic hemangiomas ranges from 23 to 62 years, with a mean of 47 years. Among these cases, 7 were female and 2 were male, indicating a higher occurrence among females. In the table, 4 patients were asymptomatic, and their lesions were discovered during physical examinations. There were 3 patients had abdominal pain, likely due to a mass pressing on the adjacent structures. In 2 patients, tumor rupture resulted in abdominal hemorrhage. Most multilocular cystic hemangiomas are large. In the data we collected, the diameter exceeded 3.4 cm, with the largest diameter measuring 18 cm and an average length of approximately 8.7 cm. Some studies classify hepatic hemangiomas larger than 3 cm or 4 cm as “giant hemangiomas,” which are more likely to produce symptoms and exhibit characteristics of atypical hemangiomas.^[10]^

Based on Table 1, the pathological and imaging features of multilocular cystic hemangiomas are as follows.

1. It is typically a single cystic solid lesion located in the liver, and is rare.
2. The lesion appeared as a multilocular cystic mass, with well-defined boundaries and lobulated edges. The cystic portion lacks enhancement, whereas the cyst wall and septa show progressive enhancement with no enhanced nodules on the cyst wall. The mass may also exhibit calcification or bleeding.
3. On a cut section of a gross specimen, the tumor macroscopically consists of multicystic loculi separated by multiple septa with variable thickness.And focal compartments may display a star-shaped pattern containing necrotic material, serous fluid, or thrombus within the capsule.The capsule wall is lined with single or multiple layers of cubic endothelial cells.
4. According to the literature^[4,9]^ and Case 2 in this study, immunohistochemical findings included CD31+, CD34+, ERG +, D2-40 - or focal +, CK 7 -, and CK19-.

Previous studies have analyzed the pathogenesis of multilocular cystic hemangiomas of the liver. Owing to the excessive proliferation of collagen fiber components within the lesion, blood flow may be slow, leading to the narrowing and disappearance of the blood sinuses and small vessel lumens. This slow blood flow can result in the formation of small thromboses within sinuses and vessels. Subsequently, endothelial cells within the blood sinuses and small vessel lumen may swell, shed, or partially shed to varying degrees, allowing many blood cells to escape into the surrounding tissue. Thrombi formed within these blood sinuses and small blood vessels may become organized and calcification can also occur. Both organized and unorganized thrombi, along with fibrous tissue, collectively contribute to the scar tissue within the tumor. The degree of collagen fiber tissue hyperplasia and extent of intracavitary thrombosis vary, resulting in different blood flow velocities.^[1]^ We speculate that the high signal observed in the DWI sequence at the edge of the lesion in the imaging findings of the 3 cases in this study may also be a characteristic manifestation of multilocular cystic hemangioma, potentially attributable to thrombosis and necrosis within the lesion. In addition, the cystic part of multilocular cystic hemangioma is serous, thrombus or necrotic material, so the tumor shows low signal on T1WI and high signal on T2WI, and does not strengthen after enhanced scanning. The central core-like solid part showed gradual contrast enhancement on dynamic magnetic resonance imaging, but it was recognized as delayed contrast enhancement usually seen in fibrotic stroma rather than centripetal fill-in enhancement typical of haemangioma.

Due to the atypical presentation of multilocular cystic hemangioma and its non-specificity in imaging, it is often misdiagnosed as other liver diseases, especially mucinous cystic tumors (MCN), such as cystadenoma or cystadenocarcinoma of the liver. Cases 1/4/6 in the table and the pathological results of Case 1/Case 2/Case 3 surgeries in this study were previously diagnosed as MCN or hepatic cystadenomas. Mucinous cystic tumors are rare cystic tumors of the biliary system, predominantly affecting middle-aged women. In over 80% of cases, highly cellular mesenchymal tissue similar to ovarian stroma is present, and this type of stroma has been reported to be exclusive to female patients.^[11, 12]^ Although most cases are benign, up to 20% have the potential to progress to cystadenocarcinoma. Differentiating between the 2 is typically challenging preoperatively; therefore, surgical treatment is the primary approach.^[13]^ Imaging typically reveals a large, isolated, multilocular cystic lesion with well-defined boundaries, visible enhancement of the capsule wall and septum, and features such as mural nodules, varying wall thicknesses, papillary projections, and internal septations.^[4, 11, 12]^ Additionally, some studies^[14]^ have reported elevated levels of CA199, CA125, and CEA in patients with hepatic cystadenoma, which may serve as diagnostic references. However, in cases of multilocular cystic hemangioma (Table 1), the CA199, CA125, and CEA levels were not elevated.

In this study, the preoperative CT and magnetic resonance imaging diagnoses in Case 2 were biliary hamartoma. Multicystic biliary hamartoma is an extremely rare benign hepatic neoplasm characterized by a localized cystic-solid mass.^[2]^ Lian et al summarized the characteristics of multicystic biliary hamartoma as follows.

1. Typically located near the liver surface or protruding from the liver
2. A localized cystic-solid neoplasm with a honeycomb-like appearance lacks well-defined borders. The cystic components showed no enhancement, whereas the solid components showed enhancement in the arterial phase.
3. Absence of dilation in intrahepatic and extrahepatic bile ducts: cystic dilatation tubes are not connected to the biliary system.
4. Ductal structures, periductal glands, fibrous connective tissues, and normal liver parenchyma were interspersed within nodular lesions.
5. Contains bile-like materials within ducts
6. Positive immunostaining for biliary-type CKs^[15]^

In addition, multilocular cystic hemangiomas should be differentiated from hepatocellular carcinoma, liver metastasis, and non-neoplastic liver disease. Hepatocellular carcinoma may have extensive necrosis or bleeding and may present as an atypical multilocular cystic mass. Notably, even multilocular cystic hepatocellular carcinoma wall nodules showed significant reinforcement in the arterial phase, decreased reinforcement in the delayed phase, and capsule reinforcement in the delayed phase, which is typical of hepatocellular carcinoma reinforcement. In addition, hepatocellular carcinoma should be considered first for multilocular cystic masses in patients with cirrhosis.^[11, 12]^ Multilocular cystic liver metastases can be caused by extensive necrosis of the liver metastases, especially neuroendocrine tumors, melanomas, or gastrointestinal stromal tumors. Multilocular cystic hepatic metastases of ovarian or colorectal mucinous adenocarcinomas are caused by the production of large amounts of mucinous protein. Irregular strengthening of the cyst wall and septum may indicate tumor cell activity in multilocular cystic metastases. However, because these imaging findings are generally nonspecific compared to other multilocular cystic tumors, understanding their primary malignancy is of utmost importance.^[11, 12]^ Non-neoplastic hepatopathy includes hepatic abscesses, echinococcosis, and intrahepatic hematomas. Liver abscesses usually exhibit characteristic imaging features such as clusters or double rings and are associated with fever. The typical manifestation of hepatic echinococcosis is an intracapsular or spoked sign that is associated with endemic areas. Intrahepatic hematoma may be associated with trauma or iatrogenic injury.^[11, 12]^

## 4. Conclusions

A multilocular cystic hemangioma is an extremely rare condition that is difficult to differentiate from other multilocular cystic liver diseases. Notably, multilocular cystic liver disease is rarely initially diagnosed as hemangioma. By ruling out other potential differential diagnoses of multilocular cystic liver disease, suspected cases of multilocular cystic hemangioma can avoid unnecessary liver resections.

## Author contributions

**Conceptualization:** Chunmiao Liang, RiSheng Yu, Liuhong Wang.

**Data curation:** Chunmiao Liang.

**Formal analysis:** Chunmiao Liang.

**Methodology:** RiSheng Yu.

**Resources:** Ying Chen.

**Writing – original draft:** Chunmiao Liang.

**Writing – review & editing:** Chunmiao Liang, RiSheng Yu, Liuhong Wang, Ying Chen.
